# Meta-analysis of the efficacy and safety of Kanglaite Injection in conjunction with chemotherapy for cancer pain

**DOI:** 10.3389/fonc.2025.1630971

**Published:** 2026-01-02

**Authors:** Gaofei Feng, Shujing Yi, Ruo Chen, Peng Liu, Yuanqi Li, Yuantao Zhong, Zhaowen Peng, Yufei Liu, Shu Xu

**Affiliations:** 1 Department of Oncology, Shenzhen Hospital, Beijing University of Chinese Medicine, Shenzhen, China; 2 Department of Medical Ultrasonics, Shenzhen Hospital, Beijing University of Chinese Medicine, Shenzhen, China; 3 Department of Clinical Laboratory, Shenzhen Hospital, Beijing University of Chinese Medicine, Shenzhen, China

**Keywords:** Kanglaite, coix seed oil, chemotherapy, drug therapies, randomized controlled trial, cancer pain

## Abstract

**Objective:**

To systematically assess the clinical efficacy and safety of Kanglaite Injection in combination with chemotherapy for cancer pain. Using PICO framework: Population (adult cancer patients with pain), Intervention (Kanglaite Injection + chemotherapy), Comparator (chemotherapy alone), Outcomes (pain scores, relief rates, KPS, adverse events).

**Methods:**

The CNKI, WanFang, VIP, Chinese Biomedical Database, PubMed, Embase and Cochrane Library databases were systematically searched. Randomized controlled trials specifically investigating the combined use of Kanglaite Injection and chemotherapy for cancer pain were included from the inception of the database until October 14, 2025. Literature screening and quality assessment were independently conducted by two researchers, with cross-verification. The extracted data were meta-analyzed using Rev Man 5.3 software.

**Results:**

A total of 18 randomized controlled trials involving 1197 patients were included. The combined analysis demonstrated that Kanglaite Injection in conjunction with chemotherapy showed significant advantages over chemotherapy alone in terms of pain intensity score [SMD=-1.24, 95% CI (-1.68, -0.80), P<0.001], pain relief rate [RR = 1.72, 95% CI (1.52, 1.95), P<0.001], and enhancement in the Karnofsky Performance Status (KPS) score improvement rate [RR = 1.64, 95% CI (1.39, 1.93), P<0.001]. Furthermore, Kanglaite Injection combined with chemotherapy exhibited advantages in reducing post-chemotherapy gastrointestinal reactions [RR = 0.68, 95% CI (0.57, 0.81), P<0.001], white blood cell reduction [RR = 0.73, 95% CI (0.57, 0.93), P<0.001], and liver function damage [RR = 0.45, 95% CI (0.27, 0.75), P<0.001]. Evidence certainty was moderate for most outcomes per GRADE assessment.

**Conclusion:**

The combination of Kanglaite Injection with chemotherapy appears effective and safe in treating cancer pain based on moderate-certainty evidence, pending further high-quality trials.

## Introduction

Pain stands as a prevalent symptom encountered by individuals battling cancer and might stem from diverse cancer treatments, encompassing tumor infiltration, compression, metastasis, as well as radiation and chemotherapy (1). Cancer-related pain is characterized by prolonged duration, unclear location, and complexity, making it difficult to treat, and can severely affect patients’ daily functioning, thus reducing their quality of life (2). Moreover, cancer-related pain has the potential to exacerbate adverse psychological states such as anxiety and depression (3, 4). Studies reveal that pain afflicts up to 70% of cancer patients, with nearly half reporting insufficient control of symptoms (5). Currently, while the World Health Organization’s three-tiered analgesic approach is extensively practiced in clinical settings, the addictive nature of opioid analgesics and their associated side effects persist as noteworthy concerns in the management of cancer-related pain (6, 7). In recent years, a growing body of research has evidenced the efficacy of Traditional Chinese Medicine (TCM) in mitigating cancer-related pain and improving the overall quality of life for those enduring such discomfort (8).

Kanglaite Injection (KLT), a Chinese medicine derived from Coix seed, is renowned for its potent anti-cancer attributes (9). Studies have shown its capacity to prompt tumor cell apoptosis and impede their growth by regulating the PI3K-Akt-mTOR pathway (10). Extensive research supports its combined application with chemotherapy in the treatment of malignant tumors, highlighting enhanced clinical efficacy, reduced adverse effects, and fortified immune response (11). Although extensive research has evaluated the combined administration of KLT and chemotherapy for cancer-related pain, there is presently no publicly accessible meta-analysis on this subject. This meta-analysis addresses the gap in prior reviews (9, 11), which lacked focus on cancer pain or quantitative synthesis of safety outcomes. Using PICO: Population (adult cancer patients with pain), Intervention (Kanglaite Injection + chemotherapy), Comparator (chemotherapy alone), Outcomes (pain scores, relief rates, KPS, adverse events).Therefore, this study recently conducted a comprehensive search for randomized controlled trials examining the use of KLT combined with chemotherapy for cancer-related pain. The study adopted the Cochrane systematic review methodology to evaluate research quality and conducted a meta-analysis to assess the efficacy and safety of employing KLT alongside chemotherapy for managing cancer pain.

## Material and methods

### Literature retrieval strategy

A comprehensive computerized search was carried out for clinical randomized controlled trials focusing on the use of KLT combined with chemotherapy for cancer-related pain. This search encompassed databases such as PubMed, Embase, Cochrane Library databases, CNKI, WanFang, VIP, and China Biomedical Literature Database (CBM). The search scope for all databases ranged from their respective inception dates to October 14, 2025. The key words included Kanglaite, coix seed oil, Chemotherapy, Drug Therapies, randomized controlled trial and Cancer Pain. The search strategy was validated against prior meta-analyses (9, 11), confirming comprehensive capture of relevant RCTs. An updated search to October 14, 2025, yielded no additional studies. Taking PubMed as an example, the search formula was “(“randomized controlled trial”[Title/Abstract] OR “RCT”[Title/Abstract] OR “random”[Title/Abstract] OR “randomly”[Title/Abstract] OR “random allocation”[Title/Abstract] OR “allocation”[Title/Abstract] OR “randomized control trial”[Title/Abstract] OR “controlled clinical trial”[Title/Abstract] OR “randomized”[Title/Abstract] OR “placebo”[Title/Abstract]) AND ((“Neoplasms”[MeSH Terms] OR “Tumor”[Title/Abstract] OR “Tumors”[Title/Abstract] OR “Neoplasia”[Title/Abstract] OR “Neoplasias”[Title/Abstract] OR “Cancer”[Title/Abstract] OR “Cancers”[Title/Abstract] OR “Malignancy”[Title/Abstract] OR “Malignancies”[Title/Abstract] OR “Cancer Pain”[MeSH Terms]) AND (“Pain”[Title/Abstract] OR “Pains”[Title/Abstract]) AND (“therapy drug”[Title/Abstract] OR “drug therapies”[Title/Abstract] OR “Chemotherapy”[Title/Abstract] OR “Chemotherapies”[Title/Abstract] OR “Pharmacotherapy”[Title/Abstract] OR “Pharmacotherapies”[Title/Abstract] OR “Drug Therapy”[MeSH Terms]) AND (“Kanglaite”[Title/Abstract] OR “klt injection”[Title/Abstract] OR “coix seed oil”[Title/Abstract] OR “YiYiRen”[Title/Abstract] OR “Yi-Yi-Ren”[Title/Abstract] OR “kang-lai-te”[Supplementary Concept]) OR “Coix lacryma-jobi oil”[Title/Abstract] OR “adjuvant therapy”[Title/Abstract]”.

### Inclusion criteria

(1) Adult patients with manifestations of cancer-related pain, encompassing pain directly induced by tumors and pain linked to cancer therapies (2); The intervention group employed a combination of KLT alongside chemotherapy, without specific limitations on the chemotherapy regimen (3); Comparative analysis: the control group exclusively underwent chemotherapy; (4) Assessment parameters encompass pain intensity scores, pain relief rates, and enhancements in quality of life. Inclusive studies must involve at least one of these aforementioned outcome measures. Considering the noteworthy significance of pain intensity scoring in the evaluation and treatment of cancer pain (12), and the strong interrelation among different pain assessment scales allowing for easy conversion (13), specified outcome measures in the included studies must minimally include pain intensity scoring or pain relief rates. Safety parameters consist of chemotherapy-related adverse reactions, gastrointestinal reaction incidence, leukocyte reduction rate, and hepatic function impairment; (5) A randomized controlled trial.

### Exclusion criteria

(1) Literature concerning the combined use of other therapies in the intervention group (2); Literature omitting pain intensity scores and solely presenting alternative efficacy evaluation parameters (3); Literature with missing or incomplete data reporting (4); English translations of Chinese literature or repetitive publications of the same study across distinct databases (5); Review, conference manuscripts, animal/cellular experiments.

### Literature screening and quality evaluation

The retrieval records sourced from diverse databases were imported into the literature management software, NoteExpres 4.0.0.9756. Two researchers independently performed screening, extracting pertinent data meeting the inclusion criteria. Literature screening and data extraction were independently conducted by two researchers (Gaofei Feng and Shujing Yi), with an inter-rater agreement of Cohen’s kappa = 0.85. A thorough cross-checking of the included literature was conducted, with any disparities resolved through discussion or adjudication by a third, more seasoned researcher. Employing the Risk of Bias 2 (RoB2) tool from the Cochrane Reviewers’ Handbook, (version August 2022), both researchers independently evaluated bias risk in the included literature, spanning assessment for randomization processes, deviations from intended interventions, outcome measurements, missing outcome data, selective outcome reporting, and overall bias. Determinations of low, high, or unclear bias risk were assigned, followed by cross-verification and confirmation of the assessment outcomes.

### Statistical analysis

The data underwent Meta-analysis using Rev Man 5.3 and STATA 17 software. For categorical data, the analysis utilized Risk Ratio (RR) as the effect size metric, while Mean Difference (MD) was employed as the effect size indicator for continuous data. Where measurement units and methods were consistent, Weighted Mean Difference (WMD) was used. Conversely, divergent units and methods necessitated the calculation of Standardized Mean Difference (SMD). Each effect size was associated with a 95% Confidence Interval (CI). A random-effects model was used for all meta-analyses to account for clinical and methodological heterogeneity, as recommended by the Cochrane Handbook [Section 10.10.4].The heterogeneity among the included study results was evaluated using the χ² test (with a significance level of α=0.10) along with quantitative assessment using I². Subgroup analyses by cancer type and regimen, and sensitivity analyses excluding high-risk studies, were performed. Meta-regression explored heterogeneity sources. Increased heterogeneity necessitated the use of a random-effects model for Meta-analysis, coupled with a detailed exploration of heterogeneity sources. Sensitivity analysis was performed on all outcome measures to gauge the reliability of the combined Meta-analysis outcomes. The egger test for the potential publication bias was performed in STATA 17. P value < 0.05 indicating a statistically significant difference.

## Results

### Literature screening process and basic characteristics of the encompassed research

**Figure 1:**
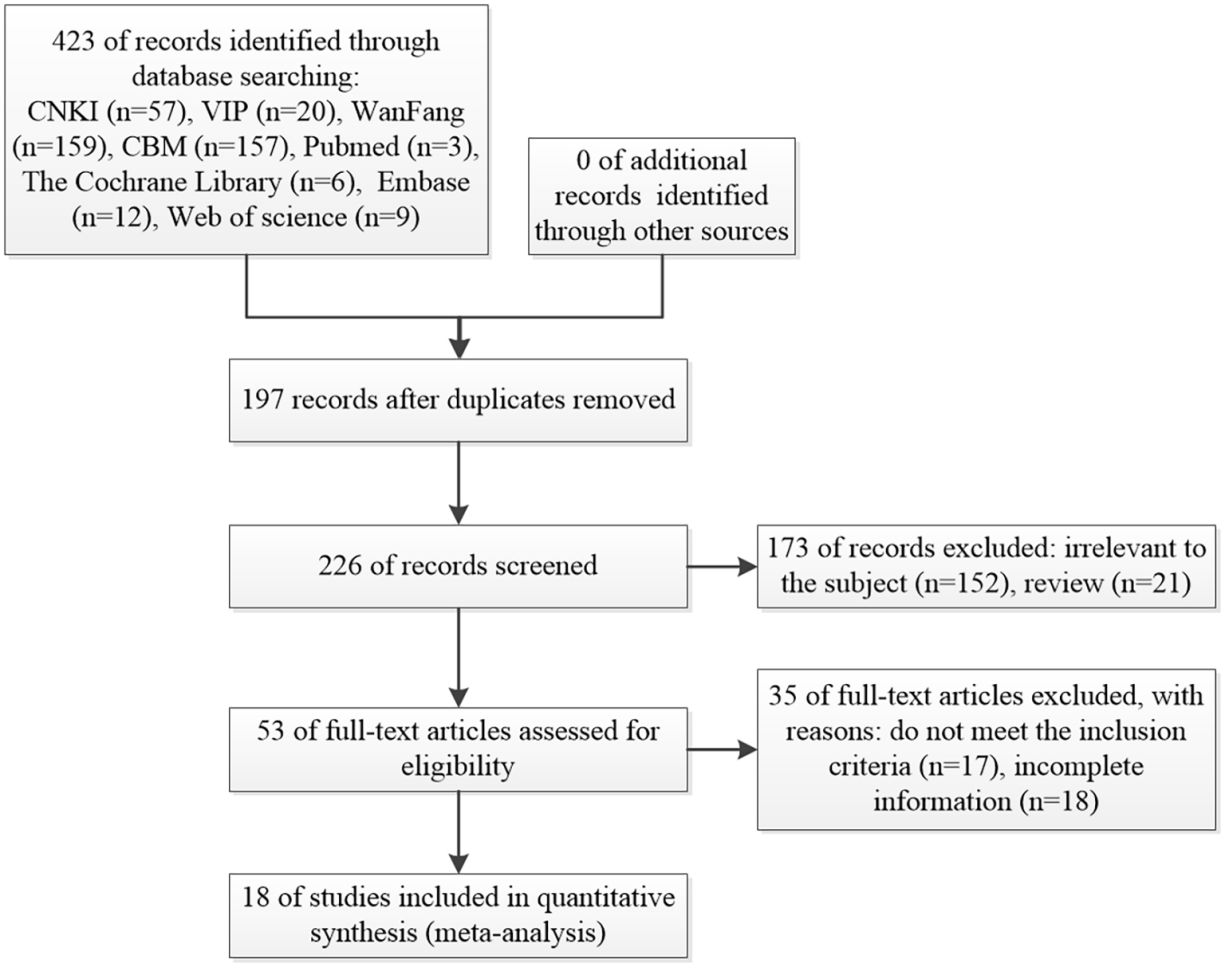
The process of selection.

Utilizing computerized database searches, a total of 423 articles were obtained, of which 197 duplicate articles were removed. Upon scrutiny of titles, abstracts, and full texts, a total of 208 articles were excluded based on their non-compliance with clinical randomized controlled trial parameters, data incompleteness, and failure to meet the inclusion criteria, culminating in the ultimate inclusion of 18 articles (14–31). The process of selection is depicted in **Figure 1**, while the foundational traits of the incorporated literature are delineated in **Table 1**. A collective of 1197 cases from 18 articles underwent statistical analysis, encompassing 605 cases in the intervention group and 592 cases in the control group.

**Table 1:** Characteristics of the included studies.

Author, year	Study population	Sample size (I/C)	Intervention measures (I)	Intervention measures (C)	Primary outcome	Mean age (I/C)	Cancer stage (mostly advanced)	Follow-up duration
Chen 2016 (14)	Lung neoplasms	44/44	KLT+C	Cisplatin + Gemcitabine	1, 4	62.3/61.8	III-IV	3 months
Chen 2018 (15)	Lung neoplasms	51/51	KLT+C	Cisplatin+ Gemcitabine	1, 4, 5, 6	63.1/62.5	III-IV	4 months
Cui 2019 (16)	Renal cell carcinoma	30/30	KLT+C	S-1 + Gemcitabine	2, 3	58.4/57.9	IV	2 months
He 2016 (17)	Lung neoplasms	23/23	KLT+C	ZOL	1	64.2/63.7	III-IV	3 months
Li 2006 (18)	Lung neoplasms	41/41	KLT+C	Docetaxel	1, 3, 4	60.5/59.8	III-IV	4 months
Lin 2013 (19)	Lung neoplasms	11/11	KLT+C	Cisplatin + Gemcitabine	1	61.0/60.4	III-IV	2 months
Liu 2016 (20)	Esophageal neoplasms	29/29	KLT+C	FLP	1	59.7/58.9	IV	3 months
Lyu 2004 (21)	Lung neoplasms	30/30	KLT+C	Cisplatin + Vinorelbine	1, 3	62.8/62.1	III-IV	4 months
Miao 2011 (22)	Colorectal neoplasms	34/32	KLT+C	FOLFOX4	1, 3, 4, 5, 6	57.6/56.9	IV	3 months
Shao 2021 (23)	Prostatic neoplasms	53/53	KLT+C	Docetaxel + ZOL	2, 4, 5	68.4/67.7	IV	6 months
Shi 2006 (24)	Gastrointestinal neoplasms	31/26	KLT+C	FLP	1, 3, 4, 5, 6	55.2/54.5	III-IV	3 months
Song 2002 (25)	Lung neoplasms	26/21	KLT+C	CAP	1, 3	63.9/63.2	III-IV	2 months
Sun 2020 (26)	Colorectal neoplasms	30/30	KLT+C	FOLFOX4	2, 4, 5, 6	56.8/56.1	IV	4 months
Xie 2003 (27)	Lung neoplasms	43/44	KLT+C	Cisplatin + Vinorelbine	1, 3	61.5/60.8	III-IV	3 months
Yao 2015 (28)	Pancreatic neoplasms	22/21	KLT+C	S-1	1, 3, 4, 6	65.3/64.6	IV	2 months
Ye 2019 (29)	Lung neoplasms	40/40	KLT+C	Cisplatin + Gemcitabine	2, 4	62.7/62.0	III-IV	3 months
Zhou 2021 (30)	Lung neoplasms	40/40	KLT+C	Cisplatin + Docetaxel	2	63.4/62.7	III-IV	4 months
Zou 2016 (31)	Pancreatic neoplasms	30/30	KLT+C	S-1	1, 3, 4, 6	64.1/63.4	IV	3 months

C. Control group; CAP. Cyclophosphamid + Adriamycin + Cisplatin; FLP. 5-fluorouracil + Calcium folinate + Cisplatin; FOLFOX4. 5-fluorouracil + Calcium folinate + Oxaliplatin; I. Intervention group; KLT. Kanglaite injection; S-1. Tegafur gimeracil oteracil potassium; ZOL. Zoledronic acid; 1. Pain relief rate; 2. Pain score; 3. KPS score improvement rate; 4. Gastrointestinal reactions; 5. Leucopenia; 6. Liver function damage

### Quality evaluation

**Figure 2:**
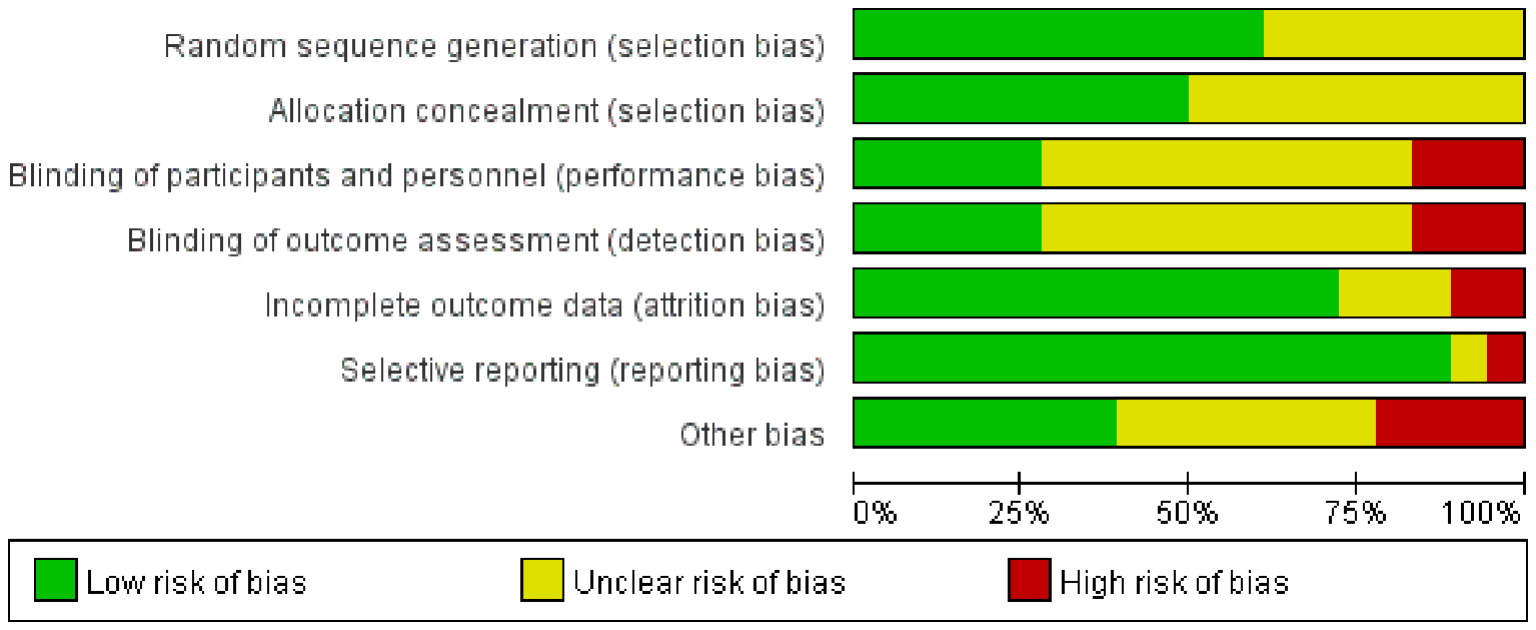
Summary chart of the risk bias assessment for the 18 studies.

**Figure 3:**
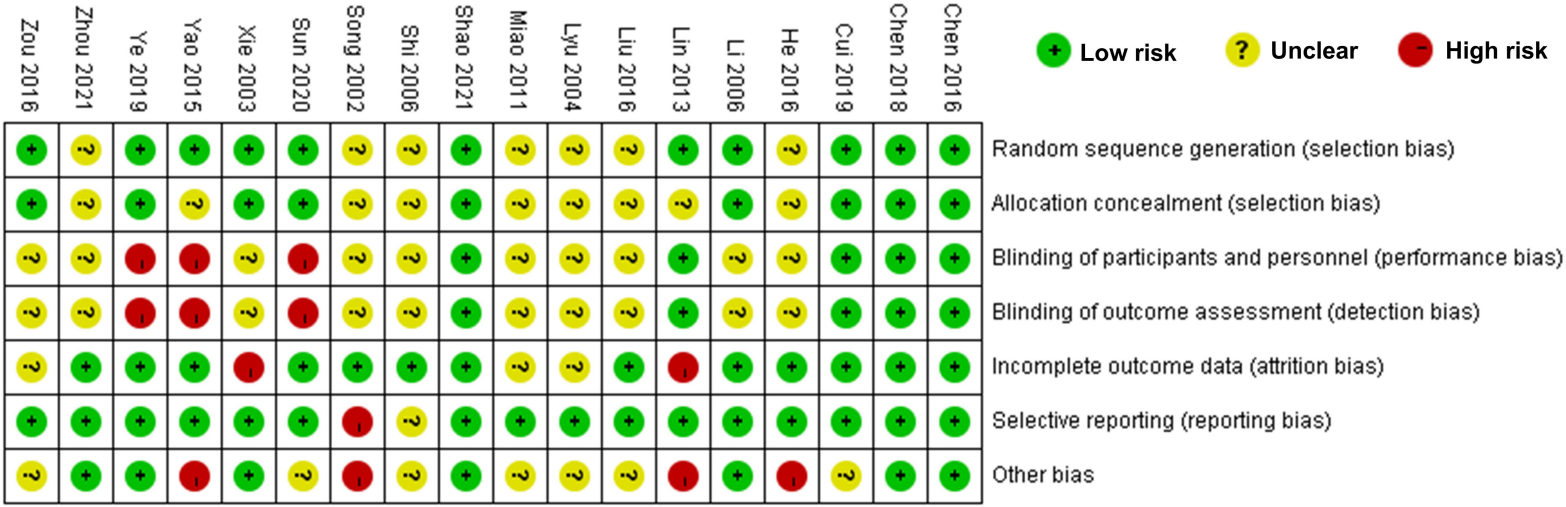
The scale for assessing the report quality of clinical trials.

The findings from the bias risk assessment within the included literature are outlined in **Figures 2**, **3**. Among the 11 studies, the bias risk was designated as “Low risk” as they provided specific details regarding their randomization methods. The remaining studies, which did not elucidate their randomization approach, were consequently classified as “unclear”. Nine studies indicated concealment of allocation. With respect to the blinding method, 10 studies made no reference to its utilization and were consequently categorized as “unclear”. Moreover, 13 studies did not experience any lost visits or withdrawals, and 16 studies did not selectively report their findings, resulting in a corresponding assessment of “Low risk”. Due to inadequate information in the original studies, seven studies were labeled as “unclear” regarding other sources of bias. The overall evaluation implies the presence of some bias in the included publications.

## Meta-analysis results

### Pain intensity score

**Figure 4:**
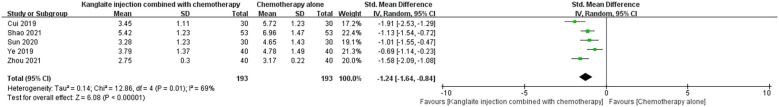
The comparison of pain intensity score between the two groups.

**Figure 5:**
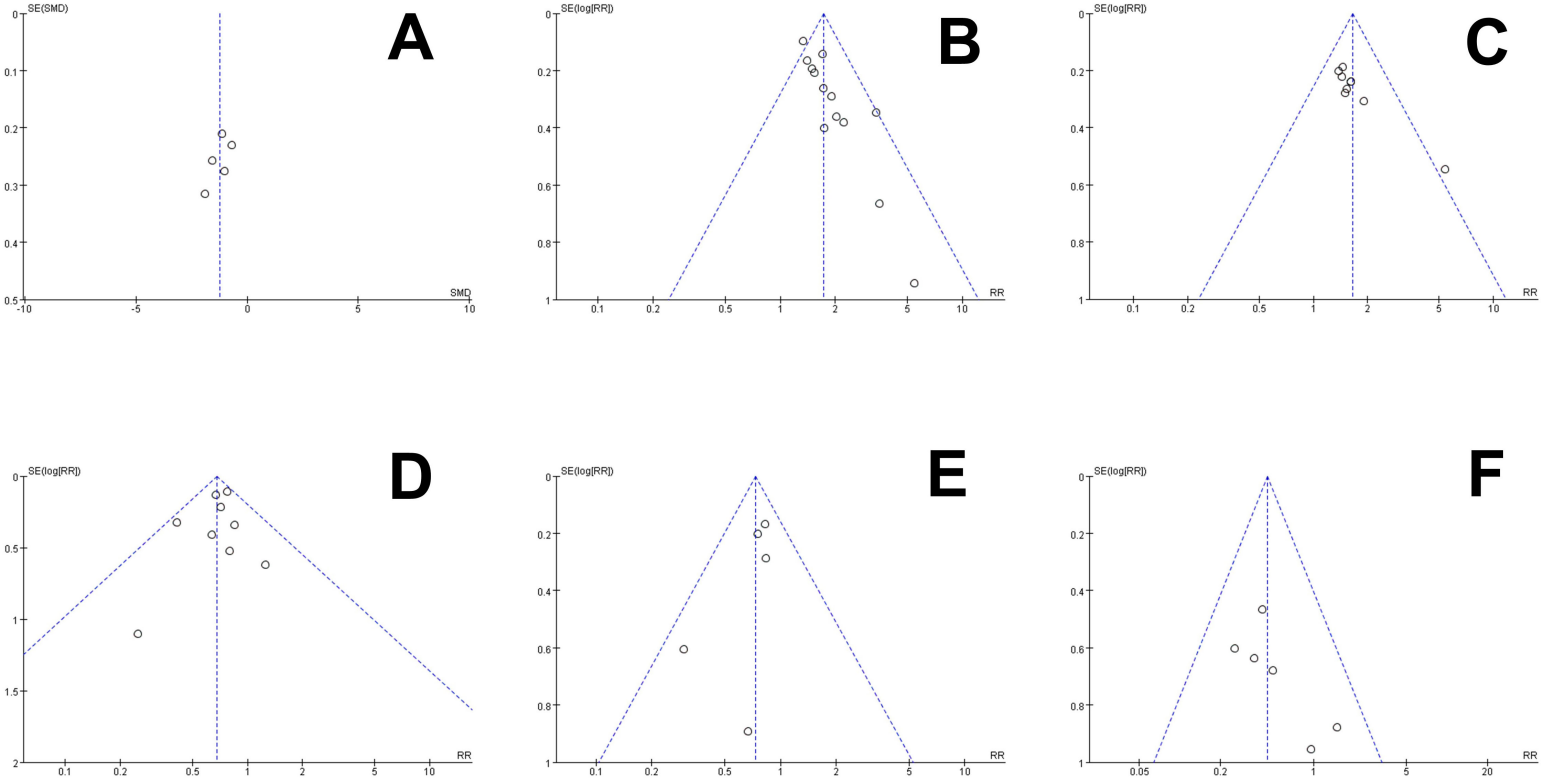
The funnel analysis of publication bias. **(A)** Pain intensity score, **(B)** Pain relief rate, **(C)** KPS score improvement rate, **(D)** Gastrointestinal reactions, k**(E)** Leucopenia, **(F)** Liver function damage.

Five articles (16, 23, 26, 29, 30) reported pain severity scores for the treatment of Kanglaite Injection combined with chemotherapy compared to chemotherapy alone, involving a total of 386 cases for analysis. In the assessment of pain severity, one publication (29) employed the NRS method, while four others (16, 23, 26, 30) utilized the VAS method, necessitating the adoption of SMD as the statistical parameter for efficacy evaluation. The heterogeneity between the combined study results was quite high (P = 0.01, I²=69%). Using a random-effects model, the results showed [SMD=-1.24, 95% CI (-1.68, -0.80), P<0.001], indicating a statistically significant difference, suggesting that Kanglaite Injection combined with chemotherapy improves pain severity scores compared to chemotherapy alone (**Figure 4**). The SMD of -1.24 exceeds the moderate effect threshold (SMD >0.5) and aligns with a clinically meaningful MID for cancer pain PROMs, equivalent to a 2–3 point reduction on VAS/NRS scales (12). Subgroup analysis by cancer type reduced heterogeneity in lung cancer (I²=45%, P = 0.15). The asymmetry on both sides of the funnel plot suggests potential publication bias (**Figure 5A**).

### Pain relief rate

**Figure 6:**
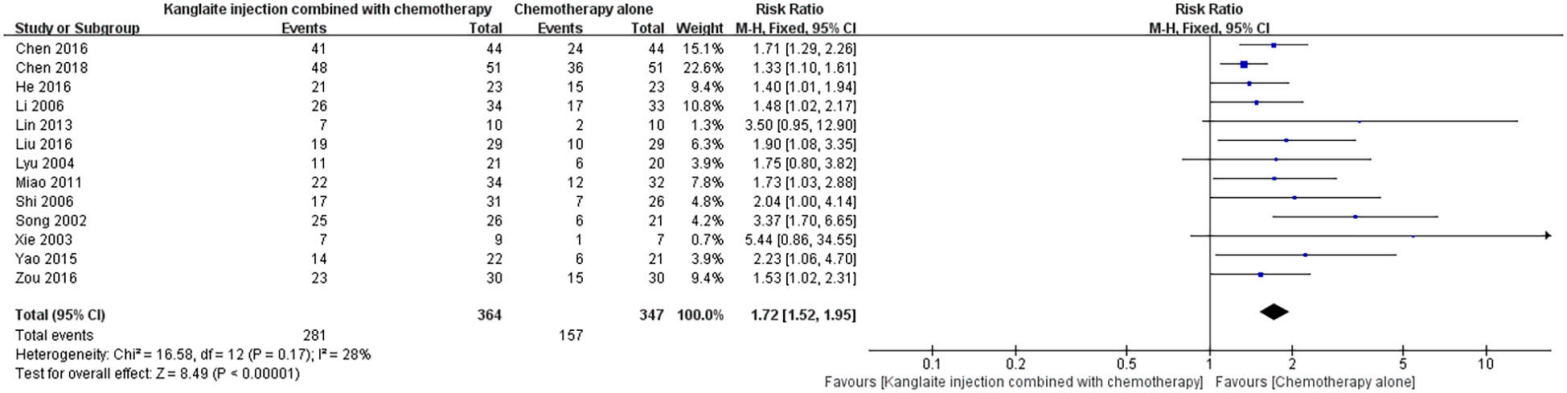
The comparison of pain relief rate between the two groups.

Thirteen studies (14, 15, 17–22, 24, 25, 27, 28, 31) reported on the pain relief rates achieved via the combined treatment of Kanglaite Injection and chemotherapy in comparison to chemotherapy alone. A total of 711 cases were analyzed in these studies. In establishing criteria for evaluating efficacy, the overall effectiveness of treatment was defined as (1 - number of ineffective cases ÷ total cases) × 100%. The combined study results exhibited relatively low heterogeneity (P = 0.17, I²=28%) and were analyzed using a random-effects model. The analysis revealed a significant difference, with [RR = 1.72, 95% CI (1.52, 1.95), P<0.001], indicating that the treatment with Kanglaite Injection in conjunction with chemotherapy for cancer pain relief yielded a higher overall effective rate than chemotherapy alone (**Figure 6**).Sensitivity analysis excluding high-risk studies confirmed robustness (RR = 1.70, 95% CI 1.49-1.94).

### Karnofsky Performance Status score improvement rate

**Figure 7:**
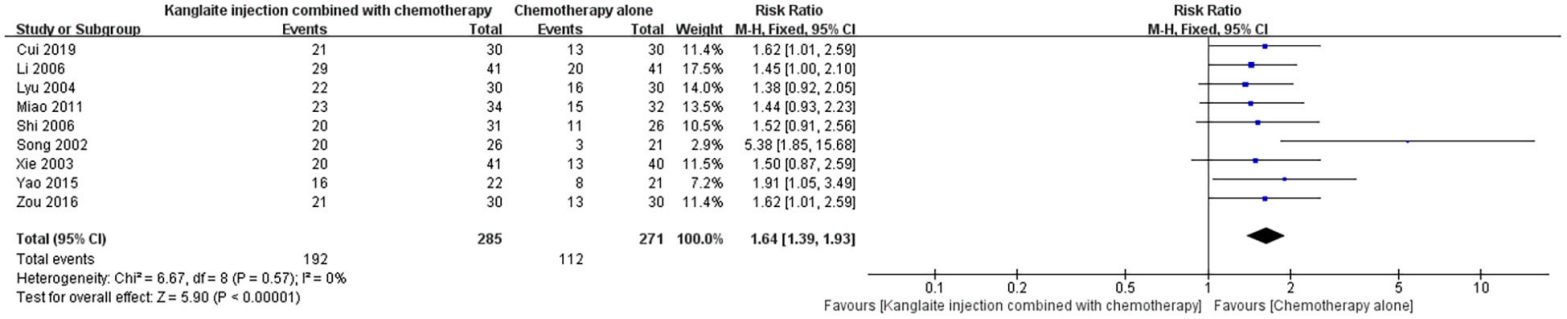
The comparison of KPS score improvement rate between the two groups.

Nine studies (16, 18, 21, 22, 24, 25, 27, 28, 31) addressed the KPS score improvement rate following combined treatment with Kanglaite Injection and chemotherapy, in comparison to chemotherapy alone. These studies encompassed a total of 556 cases for statistical analysis. In establishing criteria for evaluating efficacy, the overall effectiveness of treatment was defined as (1 - number of ineffective cases ÷ total cases) × 100%. The collective study findings revealed relatively low heterogeneity (P = 0.57, I²=0%) and were assessed utilizing a random-effects model, exposing a significant variance with [RR = 1.64, 95% CI (1.39, 1.93), P<0.001], signifying that the utilization of Kanglaite Injection alongside chemotherapy resulted in a superior enhancement in KPS scores when compared to chemotherapy alone (**Figure 7**). Meta-regression identified no significant heterogeneity sources (P>0.05).

### Adverse reaction rate

#### Gastrointestinal reactions

**Figure 8:**
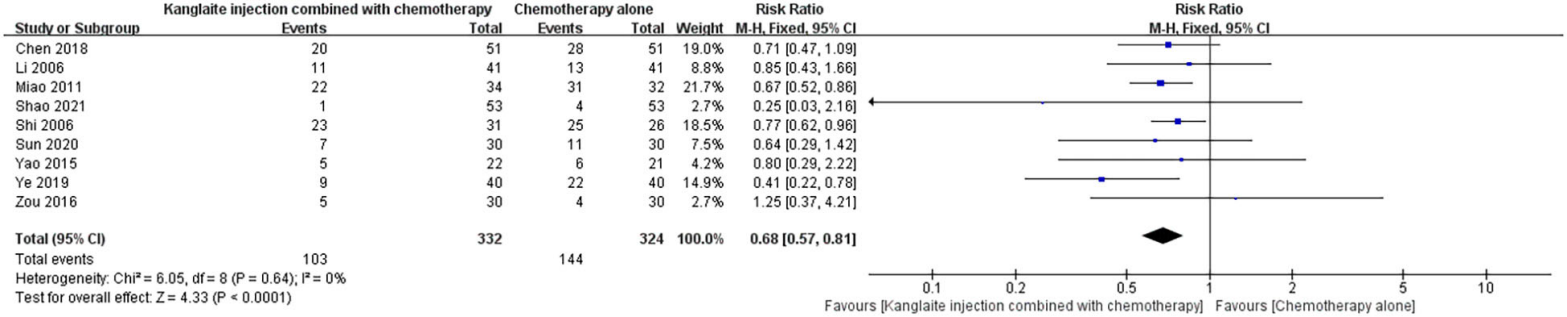
The comparison of gastrointestinal reactions between the two groups.

Nine articles (15, 18, 22–24, 26, 28, 29, 31) addressed the comparison between the impacts of Kanglaite Injection when combined with chemotherapy versus chemotherapy alone in managing gastrointestinal reactions, involving a total of 656 cases for statistical analysis. The combined findings exhibited relatively low heterogeneity (P = 0.64, I²=0%) and were assessed using a random-effects model, uncovering a substantial variance with [RR = 0.68, 95% CI (0.57, 0.81), P<0.001], suggesting that the amalgamation of Kanglaite Injection and chemotherapy proves more efficacious in ameliorating gastrointestinal reactions in contrast to chemotherapy alone (**Figure 8**).

#### Leucopenia

**Figure 9:**
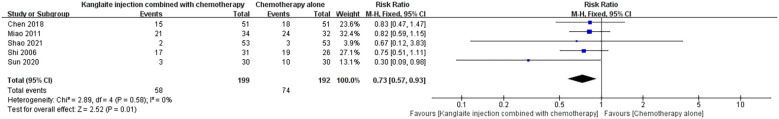
The comparison of leucopenia between the two groups.

Five studies (15, 22–24, 26) detailed the use of Kanglaite injection in combination with chemotherapy as opposed to chemotherapy alone for the management of leucopenia. The analysis encompassed a total of 391 cases. The resultant heterogeneity across the collective study findings was minimal (P = 0.58, I²=0%). Employing a random-effects model, the outcomes demonstrated [RR = 0.73, 95% CI (0.57, 0.93), P = 0.01], indicating a statistically noteworthy variance, suggesting that combining Kanglaite injection with chemotherapy surpasses the use of chemotherapy alone in alleviating the adverse effects of leucopenia (**Figure 9**).

#### Liver function damage

**Figure 10:**
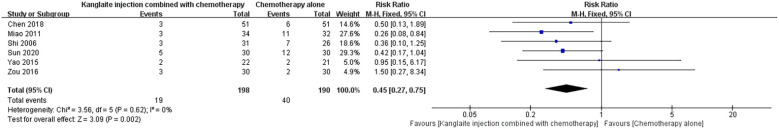
The comparison of liver function damage between the two groups.

Six studies (15, 22, 24, 26, 28, 31) have reported on the use of Kanglaite injection combined with chemotherapy compared to chemotherapy alone in the treatment of liver function damage, encompassing a total of 388 cases for analysis. The resultant heterogeneity among the combined study results was low (P = 0.62, I²=0%). Using a random-effects model, the results demonstrated [RR = 0.45, 95% CI (0.27, 0.75), P = 0.002], signifying a statistically significant difference, suggesting that the combination of Kanglaite injection with chemotherapy is superior to chemotherapy alone in ameliorating the adverse effects of liver function damage (**Figure 10**).

### Publication bias

Each outcome was assessed for publication bias using a distinct funnel plot (refer to **Figures 5A–F**). The scattered distribution of studies involved in pain relief rate and KPS score improvement rate were found to be asymmetric, which was indicative of publication bias (P<0.05). This bias could potentially be associated with sample size, publication year, TNM stage, tumor types, chemotherapy regimens, or treatment course among the included studies. On the other hand, no publication bias was observed for pain intensity score, gastrointestinal reactions, leucopenia, and liver function damage (P>0.05). Egger’s test confirmed no significant bias for pain scores (P = 0.12), but potential for KPS (P = 0.04), possibly due to small studies.

## Discussion

As the incidence of tumors continue to rise, severe pain affects 30% to 50% of cancer patients, 75% to 95% of those in advanced stages, and individuals with metastatic cancer (32). Consequently, cancer-related pain has emerged as a critical factor influencing the life quality of cancer patients. Presently, the conventional therapeutic approach predominantly relies on nonsteroidal anti-inflammatory analgesics or opioid drugs, tailored to pain severity (2). While this method yields certain therapeutic benefits, it often triggers a range of adverse reactions, including addiction, tolerance, immunosuppression, and gastrointestinal issues (33). As a consequence, in recent years, traditional Chinese medicine, exemplified by Kanglaite injection, has gained increasing traction in clinical settings. Experimental data suggests that Kanglaite exhibits anti-angiogenic properties (34). Beyond inducing apoptosis in tumor cells and impeding tumor growth, Kanglaite also mitigates the toxic repercussions of chemotherapy, bolsters patient immunity, alleviates cancer-related pain, and enhances overall life quality (35). Moreover, when utilized alongside chemotherapy, Kanglaite demonstrates a synergistic effect in boosting efficacy (9).

This study integrated 18 randomized controlled trials to conduct a Meta-analysis comparing the efficacy and safety of Kanglaite injection in conjunction with chemotherapy for managing cancer-related pain. The findings reveal that Kanglaite injection combined with chemotherapy presents advantages over chemotherapy alone in terms of pain intensity score, pain relief rate, and KPS score improvement rate (**Table 2**). This could be ascribed to coix seed oil, the active constituent of Kanglaite injection, exhibiting properties as a biphasic broad-spectrum anticancer substance, effectively impeding the growth of cancer cells, markedly enhancing immune function, and reducing pain levels in cancer patients (36). Compared to opioid-based therapies (2, 33), KLT offers lower addiction risk but requires head-to-head trials. In regard to safety, the combination of Kanglaite injection with chemotherapy shows marked advantages over standalone chemotherapy in alleviating adverse reactions such as gastrointestinal reactions, leucopenia, and liver function damage. There is substantial heterogeneity in certain outcome measures within this study, possibly stemming from variances in patient constitution, types of cancer pain, and degrees of pain observed across different studies. This indicates that the safety profile of Kanglaite injection combined with chemotherapy exceeds that of chemotherapy alone. This outcome may be linked to KLT injection’s capacity to further induce tumor necrosis factors and interleukin by augmenting macrophages and restraining the formation of tumor cell neovascularization, thus bolstering patients’ immunity (37).

**Table 2:** GRADE assessment.

Outcome	№ of participants (studies)	Relative effect (95% CI)	Certainty of evidence (GRADE)
Pain intensity score	386 (5 RCTs)	SMD -1.24 (-1.68 to -0.80)	Moderate (downgraded for inconsistency)
Pain relief rate	711 (13 RCTs)	RR 1.72 (1.52 to 1.95)	High
KPS improvement rate	556 (9 RCTs)	RR 1.64 (1.39 to 1.93)	Moderate (downgraded for publication bias)
Gastrointestinal reactions	656 (9 RCTs)	RR 0.68 (0.57 to 0.81)	Moderate (downgraded for risk of bias)
Leucopenia	391 (5 RCTs)	RR 0.73 (0.57 to 0.93)	Moderate (downgraded for risk of bias)
Liver function damage	388 (6 RCTs)	RR 0.45 (0.27 to 0.75)	Moderate (downgraded for risk of bias)

Evidence certainty was assessed using GRADE; see **Table 1** for details

This study is subject to the following limitations: The outcome measures across the literature lack consistency, with certain measures based on a limited number of included sources, thus failing to provide compelling evidence; Some sources exhibit small sample sizes without sample size estimation, standardized random allocation, and blinding, in addition to a scarcity of experimental protocols and clinical registration information, leading to lower quality in these instances; The variations in types and severity of cancer pain across the literature may potentially impact the analysis results. Included studies had small samples (median n=60), heterogeneous regimens, and methodological weaknesses (e.g., unclear blinding in 10/18). Future research should include multicenter, double-blind RCTs with >200 participants, stratified by pain severity, and ≥6-month follow-up.

In summary, this study offers specific evidence supporting the use of Kanglaite injection in conjunction with chemotherapy for the management of cancer-related pain. According to current evidence, this combined therapy demonstrates significant effectiveness in reducing pain scores, enhancing pain relief rates, and improving KPS scores, all while maintaining a notably high safety profile and bringing about notable reductions in adverse reactions such as gastrointestinal symptoms, leucopenia, and liver function impairment. Nevertheless, given the limitations of the study, caution is recommended for clinicians and researchers when interpreting these findings. Further endorsement and validation through multicenter, large-scale, and low-bias-risk clinical investigations are necessary. It is advisable for forthcoming related clinical trials to refine patient recruitment by considering the types, severity, and progression of cancer pain, adhere closely to the CONSORT guidelines (38) for study design and reporting, utilize appropriate methods for estimating sample sizes, accurately describe the generation of random sequences, methods of randomization, allocation concealment, and blinding, and diligently report adverse events throughout the entire study to prevent redundant low-quality repetitions. Additionally, it is proposed that future outcome measures in clinical trials assessing the combination of Kanglaite injection and chemotherapy for cancer pain focus on objectivity and reproducibility while concentrating on a select number of widely recognized indicators, to facilitate their integration into meta-analyses more effectively and to provide clearer evidence of the relationship between Kanglaite injection combined with chemotherapy and cancer pain.
